# Relative deprivation and physical activity participation among university students: the mediating role of psychological capital

**DOI:** 10.3389/fpsyg.2026.1923570

**Published:** 2026-10-21

**Authors:** Runqiang Dong, Mohd Sahandri Gani Bin Hamzah, Mohd Mahzan Bin Awang, Jianguo Qiu, Geng Yang

**Affiliations:** 1 Faculty of Education, Universiti Kebangsaan Malaysia, Bangi, Selangor Darul Ehsan, Malaysia; 2 School of Physical Education, Ludong University, Yantai, Shandong, China; 3 School of Physical Education, Pingxiang University, Pingxiang, Jiangxi, China

**Keywords:** mediation analysis, physical activity participation, psychological capital, relative deprivation, university students

## Abstract

**Background:**

Physical activity participation is an important component of health promotion among university students. However, students’ engagement in physical activity may be shaped not only by exercise-related conditions but also by social comparison experiences and individual psychological resources. Relative deprivation, as a negative social comparison experience, may be associated with lower physical activity participation, whereas psychological capital may represent an important psychological resource involved in this association.

**Objective:**

This study examined the relationships among relative deprivation, psychological capital, and physical activity participation among university students and examined whether psychological capital statistically mediated the association between relative deprivation and physical activity participation.

**Methods:**

A cross-sectional questionnaire survey was conducted among university students at a university in Jiangsu Province, China, in February 2026. A total of 419 valid questionnaires were included in the analysis. Physical activity participation, personal relative deprivation, and psychological capital were measured using the Physical Activity Participation Scale, the Personal Relative Deprivation Scale, and the short version of the Psychological Capital Questionnaire, respectively. Descriptive statistics, independent-samples t-tests, Pearson correlation analysis, confirmatory factor analysis, structural equation modeling, and bootstrap mediation analysis were performed.

**Results:**

The structural model showed good fit to the data (χ^2^/df = 1.343, GFI = 0.941, CFI = 0.983, TLI = 0.981, RMSEA = 0.029, SRMR = 0.040). Relative deprivation was negatively associated with physical activity participation (β = −0.391, *p* < 0.001) and psychological capital (β = −0.518, *p* < 0.001), whereas psychological capital was positively associated with physical activity participation (β = 0.328, *p* < 0.001). Bootstrap mediation analysis indicated a significant indirect association through psychological capital, suggesting a partial mediating role of psychological capital (indirect effect = −0.179, 95% CI [−0.236, −0.128]). After controlling for gender and age, the indirect effect remained significant (indirect effect = −0.178, 95% CI [−0.235, −0.127]), supporting the robustness of the findings.

**Conclusion:**

Relative deprivation was associated with lower physical activity participation among university students, with psychological capital showing a significant mediating role in this association. This indirect association remained significant after controlling for gender and age. These findings suggest that physical activity promotion in higher education should address the potential adverse role of relative deprivation and strengthen students’ psychological capital, including self-efficacy, hope, resilience, and optimism.

## 1 Introduction

Physical activity is commonly defined as any bodily movement produced by skeletal muscles that results in energy expenditure (Caspersen et al., 1985). It represents a broad category of behavior that includes structured exercise, informal exercise, incidental physical activity, sports participation, and other forms of health-related bodily movement (Stevens et al., 2020). Beyond its physiological basis, physical activity can also be understood as a context-dependent behavior shaped by individual interests, emotions, beliefs, guidance, and social relationships (Piggin, 2020). The health benefits of regular physical activity have been well documented, including improvements in cardiorespiratory fitness, muscular fitness, bone health, cardiometabolic health, and weight status (DiPietro et al., 2019; Elshahat et al., 2021; Malm et al., 2019). Evidence also suggests that some of the health benefits of physical activity during adolescence may extend into adulthood, while physical activity may also contribute to cognitive development and prosocial behavior (Warburton and Bredin, 2019). The beneficial effects of sport are largely achieved through physical activity. In addition to its direct physical health effects, sports participation may contribute to psychosocial development, personal growth, the formation of healthy lifestyles, and the maintenance of physical activity levels in later life (Piasecki et al., 2016). Regular physical activity is also associated with better mental health and may help alleviate symptoms of depression and anxiety while promoting emotional regulation and psychological adaptation (Cole, 2008; Saxena et al., 2005).

Despite these multiple health benefits, physical inactivity remains a widespread global public health problem. Recent data indicate that approximately 31% of adults and 80% of adolescents do not meet recommended levels of physical activity, and the prevalence of insufficient physical activity among adults increased by approximately five percentage points between 2010 and 2022 (Guthold et al., 2020). Physical inactivity is closely associated with an increased risk of multiple non-communicable diseases and imposes a substantial global disease and economic burden. Previous studies have estimated that insufficient physical activity contributes to millions of deaths each year and generates considerable direct healthcare costs (Ding et al., 2016; Lee et al., 2012). Therefore, physical inactivity is not merely an individual health behavior issue but has become an important global public health challenge.

University students represent a particularly important group during a transitional life stage. After entering university, students often experience substantial changes in family support, social relationships, learning environments, and daily life structures. At the same time, they face increasing academic competition, interpersonal adjustment, and pressure related to future development. Compared with secondary school students, university students no longer rely primarily on compulsory physical education classes to participate in physical activity. Instead, their physical activity participation depends more on personal initiative, self-management ability, and lifestyle organization (Perveen et al., 2018). These changes may lead some students to reduce or even discontinue regular participation in physical activity. An anonymous online survey reported that approximately 52.3% of Chinese university students had insufficient physical activity, particularly in terms of vigorous physical activity and specific types of physical activity (Xiang et al., 2020). Therefore, examining insufficient physical activity participation among university students is important not only for health promotion but also for understanding potential psychological pathways related to health-related behaviors in this population.

Among the psychological factors influencing physical activity participation among university students, relative deprivation deserves particular attention. Relative deprivation refers to a negative psychological experience that arises when individuals perceive themselves as disadvantaged in terms of resources, opportunities, status, or outcomes compared with others or with a reference group, and regard such disparities as unfair or unreasonable (Chen et al., 2025). Previous research has suggested that relative deprivation may adversely affect health-related behaviors, including physical activity, by eliciting negative emotions, reducing perceived control over life circumstances, and weakening behavioral motivation (Elgar et al., 2016). Relative deprivation has been associated with lower levels of physical activity. Studies have shown that the negative effects of relative deprivation may be related to unhealthy behavioral choices, including reduced physical exercise and imbalanced dietary behaviors. In research on relative deprivation and obesity, relative deprivation has also been found to be associated with skipping breakfast, reduced physical activity, fewer healthy dietary choices, and lower willingness to engage in weight-control behaviors (Chen et al., 2025).

From a psychological perspective, relative deprivation may promote negative cognitive processing and strong emotional responses, leading individuals to perceive their disadvantaged situation as difficult to escape. Prolonged exposure to such maladaptive cognitive and emotional states may increase the risk of depressive symptoms, which may in turn be associated with reduced exercise motivation and lower physical activity participation (Gidlow et al., 2007). Relative deprivation also implies that individuals or groups become aware of their disadvantaged position through social comparison and believe that they deserve better treatment. This perception may generate negative emotions such as anger, resentment, and anxiety, thereby impairing mental health and reducing enthusiasm for participating in collective physical activities (Fox et al., 2011).

Psychological capital refers to a positive psychological state that individuals develop during growth and adaptation, and it mainly comprises self-efficacy, hope, optimism, and resilience (Dóci et al., 2023). As an important positive psychological resource, psychological capital plays a crucial role in university students’ psychological adjustment, behavioral initiative, and maintenance of health-related behaviors. Individuals with higher levels of psychological capital generally show stronger health motivation, which may encourage them to participate more actively in physical exercise and thereby be associated with higher levels of physical activity participation (Huang et al., 2025). Previous research has also shown that psychological capital significantly predicts the quality of sports participation among individuals with disabilities and serves as a key mediator in the pathway through which social support influences sports participation, indicating that it is an important internal resource for sustaining participation in physical activity (Peng et al., 2025). Higher levels of psychological capital, including self-efficacy, resilience, hope, and optimism, may help individuals reduce negative emotions during exercise, enhance autonomy, and maintain physical exercise behavior (Xiong et al., 2025).

Meanwhile, relative deprivation may also be closely related to psychological capital. Previous studies have shown that relative deprivation, as a negative social comparison experience, may have adverse associations with individuals’ positive psychological resources. Insufficient positive psychological resources may increase individuals’ sensitivity to unfair social comparisons, thereby intensifying experiences of relative deprivation (Gao et al., 2024). Relative deprivation may also be associated with the depletion of self-regulatory resources, reducing the psychological energy required to cope with adversity and maintain positive functioning (Li C. et al., 2026). In organizational contexts, relative deprivation has been found to be associated with reduced psychological identification resources and weakened psychological capital needed to sustain positive behaviors (Han and Fan, 2025). Taken together, relative deprivation and psychological capital may represent two contrasting psychological processes, with the former reflecting perceived disadvantage and the latter representing positive psychological resources.

Although previous studies have separately examined relative deprivation, psychological capital, and physical activity participation, empirical research integrating these three constructs into a unified framework remains limited. In particular, the potential mediating pathways linking relative deprivation and physical activity participation among university students remain insufficiently understood. From the perspective of social comparison theory, university students may experience relative deprivation when they compare themselves with others in academic achievement, campus life, or developmental opportunities and perceive themselves to be disadvantaged. From the perspective of conservation of resources theory, relative deprivation may be associated with reduced psychological resources, which may further influence health-related behaviors.

Therefore, this study constructed a mediation model of relative deprivation, psychological capital, and physical activity participation among university students at a university in Jiangsu Province. Whereas previous research on university students’ physical activity has often emphasized positive factors such as social support, exercise motivation, and self-efficacy, this study focuses on the potential role of negative social comparison experiences. By incorporating relative deprivation into the explanatory framework and examining psychological capital as a potential mediating variable, this study seeks to explore whether psychological capital represents a potential explanatory pathway in the association between relative deprivation and physical activity participation. The findings may enrich evidence on relative deprivation and health-related behaviors and provide practical implications for promoting physical activity through psychological resource development.

Based on the above theoretical considerations, the following hypotheses were proposed:

H1: Relative deprivation is negatively associated with physical activity participation among university students.

H2: Relative deprivation is negatively associated with psychological capital among university students.

H3: Psychological capital is positively associated with physical activity participation among university students.

H4: Psychological capital mediates the association between relative deprivation and physical activity participation among university students.

## 2 Methodology

### 2.1 Participants

This study used a cross-sectional questionnaire survey design. Data were collected in February 2026 through offline questionnaire distribution at a university in Jiangsu Province, China. A total of 450 questionnaires were distributed. After questionnaires with incomplete responses, excessively short completion times, or patterned responses were excluded, 419 valid questionnaires were retained, yielding an effective response rate of 93.11%. All participants were undergraduate students who participated voluntarily after providing informed consent.

The final sample included 419 university students, with a mean age of 19.99 years (SD = 1.12). Of these, 205 were male (48.93%), and 214 were female (51.07%), indicating a relatively balanced gender distribution. Gender and age were used to describe the sample and were further included as basic demographic covariates in the robustness analysis.

The sample size was considered with reference to commonly used guidelines for structural equation modeling, including the participant-to-parameter ratio approach. Although power-based approaches are increasingly recommended for SEM sample size determination, a formal power or sensitivity analysis was not conducted in the present study. Given the complexity of the proposed model involving relative deprivation, psychological capital, and physical activity participation, the final sample of 419 participants was considered adequate according to commonly used SEM sample size recommendations.

## 3 Instrument

### 3.1 Physical activity participation scale

University students’ physical activity participation was measured using a Physical Activity Participation Scale developed based on the FITT principle (Zhu et al., 2025). The scale was constructed with reference to relevant content from the ACSM guidelines for exercise testing and prescription and covers four aspects of physical activity: frequency, intensity, time, and type. It consists of seven items and is used to assess the overall level of physical activity participation among university students. All items were rated on a 5-point Likert scale, with higher scores indicating higher levels of physical activity participation.

The scale showed good reliability and validity in the present sample. Cronbach’s α was 0.886, exceeding the recommended threshold of 0.70 and indicating good internal consistency. Confirmatory factor analysis showed that standardized factor loadings ranged from 0.710 to 0.741. The AVE value was 0.527, and the CR value was 0.886, indicating acceptable convergent validity (Table 1). The one-factor model also showed good fit to the data (χ^2^/df = 0.720, CFI = 1.000, TLI = 1.000, RMSEA = 0.000, SRMR = 0.014), supporting the structural validity of the scale (Table 2).

**Table 1:** Reliability and convergent validity results.

Variable	Dimension	Items	Cronbach’s α	Standardized factor loading range	AVE	CR
Physical activity participation	—	A1-A7	0.886	0.710–0.741	0.527	0.886
Relative deprivation	—	B1-B5	0.857	0.726–0.754	0.545	0.857
Psychological capital	Self-efficacy	C1-C3	0.846	0.750–0.847	0.650	0.847
	Hope	C4-C6	0.775	0.720–0.756	0.536	0.776
	Resilience	C7-C9	0.869	0.823–0.839	0.688	0.869
	Optimism	C10-C12	0.799	0.745–0.775	0.571	0.799

AVE, average variance extracted; CR, composite reliability.

**Table 2:** Model fit results of confirmatory factor analysis.

Model	χ^2^/df	CFI	TLI	RMSEA	SRMR
One-factor model of physical activity participation	0.720	1.000	1.000	0.000	0.014
One-factor model of relative deprivation	3.227	0.987	0.974	0.073	0.023
Four-factor model of psychological capital	1.288	0.995	0.993	0.026	0.022

χ^2^/df, chi-square divided by degrees of freedom; CFI, comparative fit index; TLI, Tucker–Lewis index; RMSEA, root mean square error of approximation; SRMR, standardized root mean square residual.

Although the one-factor model demonstrated exceptionally good fit indices, these results were interpreted cautiously because the scale contains a relatively small number of items and specifies a parsimonious one-factor measurement structure. Extremely high fit indices may partly reflect the simplicity of the model. Therefore, the measurement structure of physical activity participation was further examined within the overall measurement model, together with relative deprivation and the four dimensions of psychological capital. The satisfactory fit of the overall measurement model provided additional support for the adequacy of the measurement structure.

### 3.2 Personal Relative Deprivation Scale

University students’ relative deprivation was measured using the Personal Relative Deprivation Scale (PRDS) (Callan et al., 2011). The scale was developed by Callan et al. (2011) and consists of five items. It is mainly used to assess individuals’ feelings of deprivation, perceived unfairness, and dissatisfaction when comparing themselves with similar others. Items were rated on a 6-point Likert scale. Items 2 and 4 were reverse-scored before calculating the mean score across all items. Higher scores indicate higher levels of relative deprivation.

The PRDS showed good reliability and validity in the present sample. Cronbach’s α was 0.857, indicating good internal consistency. Confirmatory factor analysis showed that standardized factor loadings ranged from 0.726 to 0.754. The AVE value was 0.545, and the CR value was 0.857, indicating acceptable convergent validity (Table 1). The one-factor model achieved acceptable fit to the data (χ^2^/df = 3.227, CFI = 0.987, TLI = 0.974, RMSEA = 0.073, SRMR = 0.023), supporting the structural validity of the scale (Table 2).

### 3.3 Psychological Capital Questionnaire

Psychological capital was measured using the short version of the Psychological Capital Questionnaire, namely the Psychological Capital Questionnaire-12 (PCQ-12) (Avey et al., 2011). Based on psychological capital theory, the questionnaire includes four dimensions: self-efficacy, hope, resilience, and optimism, with a total of 12 items. In the present study, these four dimensions were modeled as first-order factors, and psychological capital was further specified as a second-order latent construct in the structural equation model.

The PCQ-12 showed good reliability and validity in the present sample. Cronbach’s α values for the four dimensions ranged from 0.775 to 0.869, exceeding the recommended threshold of 0.70. Confirmatory factor analysis showed that standardized factor loadings ranged from 0.720 to 0.847. The AVE values of the four dimensions ranged from 0.536 to 0.688, and the CR values ranged from 0.776 to 0.869, indicating acceptable convergent validity (Table 1). The four-factor model of psychological capital fitted the data well (χ^2^/df = 1.288, CFI = 0.995, TLI = 0.993, RMSEA = 0.026, SRMR = 0.022), supporting the four-dimensional measurement structure of psychological capital (Table 2). In subsequent structural equation modeling, psychological capital was modeled as a second-order latent construct reflected by these four first-order dimensions.

### 3.4 Procedure

Data were collected using paper-based questionnaires administered offline at a university in Jiangsu Province in February 2026. Before distribution, the researchers explained the purpose of the study, the survey content, and the instructions for completion. Participants were informed that participation was voluntary, that all responses would be anonymous, and that they could withdraw from the survey at any stage. No personally identifiable information was collected.

After informed consent was obtained, participants completed the questionnaires independently in classrooms or other designated campus locations. To ensure procedural consistency, all participants received the same standardized instructions. The questionnaire included measures of physical activity participation, relative deprivation, psychological capital, and demographic information. Completion took approximately 10–15 min. A total of 450 questionnaires were distributed, and 419 valid questionnaires were retained after invalid responses were excluded.

### 3.5 Data analysis

All statistical analyses were conducted using SPSSAU. Before analysis, the raw data were screened, and questionnaires with incomplete responses, excessively short completion times, or patterned answers were excluded. Univariate outliers were examined using standardized Z-scores, with |Z| > 3.29 as the criterion. Multivariate outliers were assessed using Mahalanobis distance, with *p* < 0.001 as the identification criterion. Normality was evaluated using skewness and kurtosis values. Data were considered not to seriously deviate from normality when the absolute value of skewness was less than 2, and the absolute value of kurtosis was less than 7. Multicollinearity was assessed using the variance inflation factor (VIF). Because all variables were measured using self-reported questionnaires at the same time point, Harman’s single-factor test was used to assess potential common method bias.

Internal consistency reliability was evaluated using Cronbach’s α. Convergent validity was assessed using standardized factor loadings, average variance extracted (AVE), and composite reliability (CR). Confirmatory factor analysis (CFA) was conducted to evaluate the structural validity of each scale and the fit of the overall measurement model. The Physical Activity Participation Scale and the Personal Relative Deprivation Scale were tested as one-factor structures, whereas the Psychological Capital Questionnaire was tested as a four-factor structure comprising self-efficacy, hope, resilience, and optimism. Physical activity participation, relative deprivation, self-efficacy, hope, resilience, and optimism were then included in a single overall measurement model. For CFA and SEM, the Likert-scale items were treated as approximately continuous indicators, and the models were estimated using the maximum likelihood (ML) method in SPSSAU. Discriminant validity was further examined using the heterotrait-monotrait ratio (HTMT).

Model fit was evaluated using χ^2^/df, CFI, TLI, RMSEA, and SRMR. A χ^2^/df value below 3 was considered to indicate good fit, whereas a value below 5 was considered acceptable. CFI and TLI values greater than 0.90 and RMSEA and SRMR values below 0.08 indicated acceptable model fit. Pearson correlation analysis was used to examine the relationships among the main variables. For descriptive statistics, correlation analyses, and independent-samples *t*-tests, physical activity participation, relative deprivation, and psychological capital were represented by the mean scores of their respective scale items. In contrast, confirmatory factor analysis (CFA) and structural equation modeling (SEM) were conducted using item-level indicators within a latent-variable framework. Psychological capital was specified as a second-order latent construct reflected by four first-order dimensions: self-efficacy, hope, resilience, and optimism. For model identification, the loading of optimism on the second-order psychological capital construct was fixed at 1.000 as the reference indicator, while the loadings of the other first-order dimensions were freely estimated. Independent-samples t-tests were used to compare male and female students in physical activity participation, relative deprivation, and psychological capital.

Structural equation modeling was used to examine the path relationships among relative deprivation, psychological capital, and physical activity participation. The SEM analysis was conducted using maximum likelihood estimation. Given that all constructs were measured using multi-category Likert-scale items and the data showed no serious deviations from normality, the observed indicators were treated as approximately continuous variables in the confirmatory factor analysis and structural equation modeling analyses. In the SEM model, psychological capital was specified as a second-order latent construct reflected by four first-order dimensions: self-efficacy, hope, resilience, and optimism. Bootstrap analysis with 5,000 resamples was conducted to test the mediating role of psychological capital in the association between relative deprivation and physical activity participation. To examine robustness, a covariate-adjusted mediation model was further estimated with gender and age included as control variables. The mediating effect was considered significant when the 95% confidence interval of the indirect effect did not include zero. All statistical tests used *p* < 0.05 as the criterion for statistical significance.

## 4 Findings

After data screening, 419 valid questionnaires were retained for analysis. No severe univariate outliers were detected in the main study variables, and the Mahalanobis distance test did not identify severe multivariate outliers requiring exclusion. The normality test showed that the absolute skewness values of physical activity participation, relative deprivation, and psychological capital ranged from 0.011 to 0.120, whereas the absolute kurtosis values ranged from 0.670 to 0.862. These values were within the acceptable range, indicating that the main variables did not seriously deviate from normality.

Harman’s single-factor test showed that the first factor in the unrotated factor analysis had an eigenvalue of 8.909 and explained 37.119% of the total variance. Although this value did not exceed the commonly used 40% threshold, Harman’s single-factor test alone cannot completely rule out the possibility of common method bias. Therefore, additional CFA-based comparisons were conducted to further evaluate whether common method variance substantially influenced the measurement structure. In addition, the multicollinearity test showed that all VIF values were within the acceptable range, suggesting that multicollinearity was not a concern in the present study.

### 4.1 Descriptive statistics

Table 3 presents the basic sample characteristics and descriptive statistics of the main study variables. The mean age of the participants was 19.99 years (SD = 1.12). The gender distribution was relatively balanced, with 205 male students, accounting for 48.93%, and 214 female students, accounting for 51.07%.

**Table 3:** Sample characteristics and descriptive statistics of the main variables.

Variable	M ± SD/*n*%
Age	19.99 ± 1.12
Gender	
Male	205 (48.93%)
Female	214 (51.07%)
Physical activity participation	2.94 ± 0.91
Relative deprivation	3.51 ± 0.90
Psychological capital	3.65 ± 1.04

As shown in Table 3, the mean score for physical activity participation was 2.94 (SD = 0.91), indicating a moderate level of participation. The mean score for relative deprivation was 3.51 (SD = 0.90), suggesting that participants reported a certain degree of perceived relative deprivation. The mean score for psychological capital was 3.65 (SD = 1.04), indicating a moderately high level of psychological capital. Overall, the distributions of the main variables were within a reasonable range.

### 4.2 Normality test

Normality tests were conducted to examine whether the main variables met the assumptions required for subsequent parametric analyses.

The absolute skewness values of the three variables ranged from 0.011 to 0.120, and the absolute kurtosis values ranged from 0.670 to 0.862. These values were within the acceptable range, indicating that the main variables did not seriously deviate from normality and were suitable for subsequent correlation analysis and structural equation modeling (Table 4).

**Table 4:** Normality test results of the main variables.

Variable	N	Skewness	Kurtosis
Physical activity participation	419	0.12	−0.67
Relative deprivation	419	−0.01	−0.71
Psychological capital	419	0.05	−0.86

Because all variables were measured using self-reported questionnaires at the same time point, potential common method bias (CMB) was considered. Harman’s single-factor test was first conducted as an initial diagnostic procedure. The first factor in the unrotated factor analysis had an eigenvalue of 8.909 and explained 37.119% of the total variance (Table 5). This result indicated that no single factor accounted for the majority of variance. However, given the limitations of Harman’s single-factor test, a CFA-based comparison of alternative measurement models was further conducted to provide a more rigorous assessment of potential common method bias (Table 5).

**Table 5:** Results of Harman’s single-factor test.

Factor	Eigenvalue	Variance explained (%)	Cumulative variance explained (%)
First factor	8.909	37.119	37.119

### 4.3 Measurement model testing

To examine the adequacy of the overall measurement structure, physical activity participation, relative deprivation, and psychological capital were included in the measurement model for confirmatory factor analysis. Psychological capital was specified as a second-order latent construct reflected by four first-order dimensions: self-efficacy, hope, resilience, and optimism.

As shown in Table 6, the overall measurement model showed good fit to the data: χ^2^ = 273.413, df = 237, *p* = 0.052, χ^2^/df = 1.154, GFI = 0.949, CFI = 0.993, TLI = 0.992, RMSEA = 0.019, and SRMR = 0.030. All major fit indices met the recommended criteria, indicating that the latent variable measurement structure was appropriate for subsequent correlation analysis and structural model testing.

**Table 6:** Fit indices of the overall measurement model.

Model	χ^2^	df	*p*	GFI	CFI	TLI	RMSEA	SRMR
Overall measurement model	273.413	237	0.052	0.949	0.993	0.992	0.019	0.030

χ^2,^ chi-square; df, degrees of freedom; GFI, goodness-of-fit index; CFI, comparative fit index; TLI, Tucker–Lewis index; RMSEA, root mean square error of approximation; SRMR, standardized root mean square residual.

In the second-order measurement model, Optimism was selected as the reference indicator of the Psychological Capital construct. Accordingly, the unstandardized loading of Optimism was fixed at 1.000 for model identification. The corresponding standardized loading was also 1.000 in the standardized solution. The residual variance of Optimism was not explicitly constrained to zero in the model specification; rather, the zero residual variance observed in the standardized solution resulted from the identification procedure. This boundary solution was examined, and no improper estimation or inadmissible solution was identified. The standardized loadings of the remaining freely estimated first-order dimensions were 0.789 for self-efficacy, 0.777 for hope, and 0.756 for resilience, indicating substantial contributions of these dimensions to the second-order psychological capital construct (Table 7).

**Table 7:** Standardized second-order factor loadings and identification constraint of psychological capital.

First-order dimension	Standardized loading	Identification status
Self-efficacy	0.789	Freely estimated
Hope	0.777	Freely estimated
Resilience	0.756	Freely estimated
Optimism	1.000	Reference indicator; unstandardized loading fixed at 1.000

Optimism was selected as the reference indicator for model identification. Its unstandardized loading was fixed at 1.000. The corresponding standardized loading was also 1.000 in the standardized solution. Therefore, no standard error, critical ratio, or p-value was estimated for this fixed parameter.

Discriminant validity among the constructs was further examined using the heterotrait-monotrait ratio (HTMT).

The HTMT values among the constructs ranged from 0.407 to 0.848, all below the recommended threshold of 0.90. These results indicated acceptable discriminant validity among the constructs (Table 8).

**Table 8:** HTMT results for discriminant validity.

Variable	1	2	3	4	5	6
1. Physical activity participation	–					
2. Relative deprivation	0.561	–				
3. Self-efficacy	0.416	0.407	–			
4. Hope	0.502	0.448	0.548	–		
5. Resilience	0.478	0.467	0.495	0.560	–	
6. Optimism	0.495	0.498	0.848	0.807	0.789	–

Although the HTMT value between optimism and self-efficacy was relatively high (HTMT = 0.848), it remained below the recommended threshold of 0.90, indicating acceptable discriminant validity between these two constructs. The relatively strong association between optimism and self-efficacy is theoretically understandable because both represent positive psychological resources related to individuals’ beliefs about their capabilities and expectations for future outcomes.

In addition, the correlation between optimism and overall psychological capital was relatively high (r = 0.917). This finding is expected because psychological capital was modeled as a second-order latent construct consisting of four first-order dimensions, including self-efficacy, hope, resilience, and optimism. Therefore, the strong association between optimism and psychological capital reflects the hierarchical structure of the construct rather than a lack of conceptual distinction.

To further assess potential common method bias (CMB), a confirmatory factor analysis (CFA)-based comparison was conducted. Specifically, a single-factor model, in which all observed items were loaded onto one latent factor, was compared with the proposed six-factor measurement model consisting of physical activity participation, relative deprivation, self-efficacy, hope, resilience, and optimism.

The single-factor model showed substantially poorer fit compared with the proposed six-factor measurement model, suggesting that the study constructs were empirically distinguishable and that common method variance was unlikely to fully account for the observed relationships among variables.

As shown in Table 9, the single-factor model demonstrated poor fit to the data (χ^2^ = 2108.489, df = 252, χ^2^/df = 8.367, CFI = 0.592, TLI = 0.594, RMSEA = 0.133, and SRMR = 0.110). In contrast, the proposed six-factor measurement model showed excellent fit to the data (χ^2^ = 273.413, df = 237, χ^2^/df = 1.154, CFI = 0.993, TLI = 0.992, RMSEA = 0.019, and SRMR = 0.030). The substantially better fit of the six-factor model suggested that the observed covariance among constructs was unlikely to be explained by a single common factor alone.

**Table 9:** Comparison of measurement models for assessing potential common method bias.

Model	χ^2^	df	χ^2^/df	CFI	TLI	RMSEA	SRMR
Single-factor model	2108.489	252	8.367	0.592	0.594	0.133	0.110
Six-factor measurement model	273.413	237	1.154	0.993	0.992	0.019	0.030

The single-factor model specified all observed items loading onto one latent factor. The six-factor measurement model included physical activity participation, relative deprivation, self-efficacy, hope, resilience, and optimism as separate latent constructs.

### 4.4 Correlations among study variables

Pearson correlation analysis was conducted to examine the preliminary relationships among physical activity participation, relative deprivation, and psychological capital.

Relative deprivation was significantly and negatively correlated with physical activity participation (r = −0.353, *p* < 0.01), whereas psychological capital was significantly and positively correlated with physical activity participation (r = 0.494, *p* < 0.01). Relative deprivation was also significantly and negatively correlated with psychological capital (r = −0.418, *p* < 0.01). In addition, all four dimensions of psychological capital, including self-efficacy, hope, resilience, and optimism, were positively correlated with physical activity participation and negatively correlated with relative deprivation. These results were consistent with the hypothesized directions and provided preliminary support for the mediation analysis (Table 10).

**Table 10:** Pearson correlation analysis results of the main variables.

Variable	1	2	3	4	5	6	7
1. Physical activity participation	1						
2. Relative deprivation	−0.353 ^**^	1					
3. Psychological capital	0.494 ^**^	−0.418 ^**^	1				
4. Self-efficacy	0.360 ^**^	−0.310 ^**^	0.790 ^**^	1			
5. Hope	0.454 ^**^	−0.360 ^**^	0.873 ^**^	0.673 ^**^	1		
6. Resilience	0.419 ^**^	−0.360 ^**^	0.788 ^**^	0.424^**^	0.509 ^**^	1	
7. Optimism	0.416 ^**^	−0.370 ^**^	0.917 ^**^	0.698 ^**^	0.738 ^**^	0.658 ^**^	1

**p < 0.01.

### 4.5 Preliminary analysis by gender

Independent-samples t-tests were conducted to compare male and female students in psychological capital, relative deprivation, and physical activity participation.

No significant gender differences were found in psychological capital (t = 0.582, *p* = 0.561), relative deprivation (t = −0.087, *p* = 0.931), or physical activity participation (t = 1.030, *p* = 0.303) (Table 11). Therefore, the subsequent structural model focused on the relationships among relative deprivation, psychological capital, and physical activity participation, while gender and age were included as control variables in the robustness analysis.

**Table 11:** Gender differences in main variables.

Variable	Female (*n* = 214)	Male (*n* = 205)	t	*p*
Psychological capital	3.67 ± 1.05	3.62 ± 1.04	0.582	0.561
Relative deprivation	3.50 ± 0.90	3.51 ± 0.90	−0.087	0.931
Physical activity participation	2.98 ± 0.93	2.89 ± 0.89	1.030	0.303

### 4.6 Structural relationships

Structural equation modeling was used to examine the relationships among relative deprivation, psychological capital, and physical activity participation. Relative deprivation was specified as the independent variable, physical activity participation as the dependent variable, and psychological capital as the mediating variable. Because psychological capital consists of four dimensions, namely self-efficacy, hope, resilience, and optimism, it was modeled as a second-order latent variable reflected by these four first-order dimensions.

#### 4.6.1 Model fit

The hypothesized structural model showed good fit to the data: χ^2^ = 328.920, df = 245, χ^2^/df = 1.343, GFI = 0.941, CFI = 0.983, TLI = 0.981, RMSEA = 0.029, and SRMR = 0.040. The χ^2^/df value was below 3, CFI and TLI were above 0.90, and RMSEA and SRMR were below 0.08. These results indicated that the structural model fitted the sample data well and was suitable for path analysis (Table 12).

**Table 12:** Structural model fit indices.

Model	χ^2^	df	χ^2^/df	GFI	CFI	TLI	RMSEA	SRMR
Structural model	328.920	245	1.343	0.941	0.983	0.981	0.029	0.040

χ^2,^ chi-square; df, degrees of freedom; GFI, goodness-of-fit index; CFI, comparative fit index; TLI, Tucker–Lewis index; RMSEA, root mean square error of approximation; SRMR, standardized root mean square residual.

#### 4.6.2 Direct path effects

All hypothesized direct paths in the structural model were statistically significant. Psychological capital was modeled as a second-order latent construct reflected by four first-order dimensions: self-efficacy, hope, resilience, and optimism. Relative deprivation was negatively associated with physical activity participation (B = −0.316, SE = 0.050, CR = −6.301, *p* < 0.001; standardized β = −0.391), indicating that higher relative deprivation was associated with lower physical activity participation; thus, H1 was supported. Relative deprivation was also negatively associated with psychological capital (B = −0.576, SE = 0.065, CR = −8.794, *p* < 0.001; standardized β = −0.518), supporting H2. In addition, psychological capital was positively associated with physical activity participation (B = 0.238, SE = 0.044, CR = 5.450, *p* < 0.001; standardized β = 0.328), supporting H3 (Table 13). Figure 1 presents the standardized structural path coefficients among the latent variables. The direct path results from SEM provided the basis for subsequently examining the indirect association of psychological capital through bootstrap mediation analysis.

**Table 13:** Direct effects in the structural model.

**Path**	**B**	**SE**	**z/CR**	***p*-value**	**Standardized β**	**Result**
Relative deprivation → physical activity participation	−0.316	0.050	−6.301	<0.001	−0.391	Supported
Relative deprivation → psychological capital	−0.576	0.065	−8.794	<0.001	−0.518	Supported
Psychological capital → physical activity participation	0.238	0.044	5.450	<0.001	0.328	Supported

SE, standard error; CR, critical ratio. B, unstandardized estimate; β, standardized coefficient; SE, standard error; CR, critical ratio.

**Figure 1:**
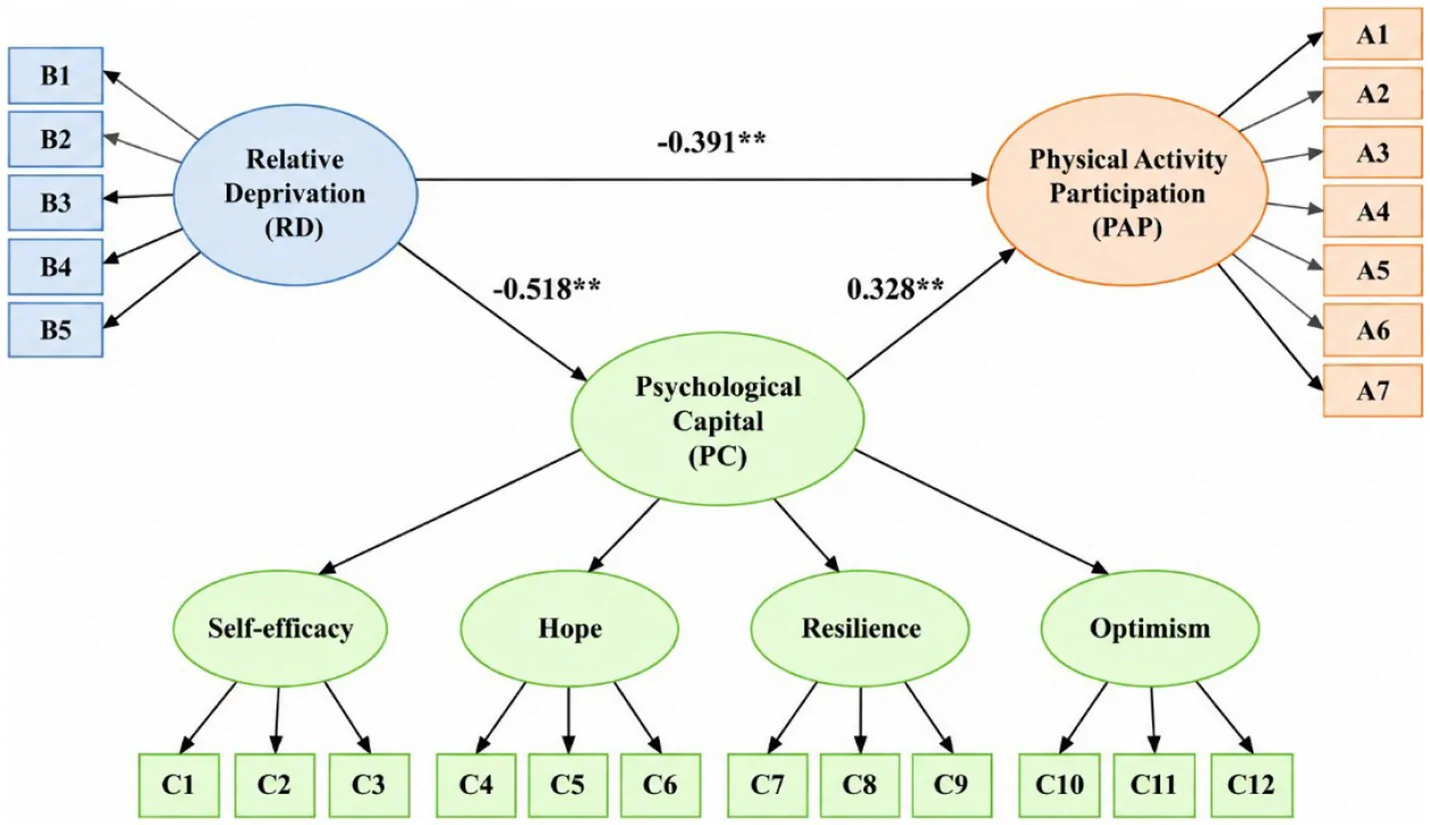
Structural model of the mediating role of psychological capital in the association between relative deprivation and physical activity participation. *Note:* Path coefficients shown in the figure are standardized structural coefficients. The loading of optimism as an indicator of the second-order psychological capital construct was fixed at 1.000 for model identification.

### 4.7 Mediation effect and robustness testing

#### 4.7.1 Mediation effect in the main model

It should be noted that the structural equation modeling and bootstrap mediation analysis were conducted using different analytical approaches. Specifically, the structural model presented in Figure 1 estimated the relationships among latent constructs, including relative deprivation, psychological capital, and physical activity participation. In contrast, the bootstrap mediation analysis was conducted using composite scores derived from the corresponding scale items to estimate the indirect effect and its confidence interval. Therefore, differences in the estimated path coefficients between the SEM model and the bootstrap mediation analysis reflect differences in model specification rather than inconsistencies in the findings. These two analytical approaches were used complementarily to examine the structural relationships and the statistical significance of the indirect association.

Following this distinction, bootstrap analysis with 5,000 resamples was conducted to further examine the indirect effect of psychological capital in the association between relative deprivation and physical activity participation. The total effect of relative deprivation on physical activity participation was significant (estimate = −0.361, *p* < 0.001), with a 95% bootstrap CI of [−0.453, −0.269]. After psychological capital was included in the model, the direct effect remained significant (estimate = −0.182, *p* < 0.001), with a 95% bootstrap CI of [−0.274, −0.089]. The indirect effect through psychological capital was also significant (estimate = −0.179, Boot SE = 0.027, *p* < 0.001), with a 95% bootstrap CI of [−0.236, −0.128] (Table 14).

**Table 14:** Total, direct, and indirect effects in the mediation model.

Effect	Path	Effect value	SE/Boot SE	95% Bootstrap CI	p-value
Total effect	Relative deprivation → Physical activity participation	−0.361	0.047	[−0.453, −0.269]	<0.001
Direct effect	Relative deprivation → Physical activity participation	−0.182	0.047	[−0.274, −0.089]	<0.001
Indirect effect	Relative deprivation → Psychological capital → Physical activity participation	−0.179	0.027	[−0.236, −0.128]	<0.001

SE, standard error; Boot SE, Bootstrap standard error. For the total and direct effects, SE refers to the standard error of the effect estimate; for the indirect effect, Boot SE refers to the Bootstrap standard error. The indirect effect was tested using 5,000 Bootstrap resamples. CI, confidence interval.

These results indicated that psychological capital partially mediated the association between relative deprivation and physical activity participation. In other words, relative deprivation was associated with lower physical activity participation both directly and indirectly through its association with psychological capital. Therefore, H4 was supported. These bootstrap findings complemented the SEM results by providing additional evidence for the statistical mediating role of psychological capital.

#### 4.7.2 Robustness check after controlling for gender and age

To examine the robustness of the findings, gender and age were included as control variables in the mediation model. These covariates were entered into the equations in which relative deprivation predicted psychological capital, relative deprivation predicted physical activity participation, and relative deprivation and psychological capital jointly predicted physical activity participation.

After controlling for gender and age, relative deprivation remained negatively associated with physical activity participation (β = −0.354, *p* < 0.001) and psychological capital (β = −0.415, *p* < 0.001). Psychological capital remained positively associated with physical activity participation (β = 0.421, *p* < 0.001). When psychological capital was included simultaneously, the direct association between relative deprivation and physical activity participation remained significant (β = −0.179, *p* < 0.001). These results indicated that the main path relationships were stable after controlling for basic demographic variables (Table 15).

**Table 15:** Regression results of the mediation model after controlling for gender and age.

Predictor	Model 1: physical activity participation			Model 2: psychological capital			Model 3: physical activity participation		
	B	SE	β	B	SE	β	B	SE	β
Age	0.005	0.037	0.006	−0.060	0.041	−0.065	0.027	0.034	0.034
Gender	−0.089	0.084	−0.049	−0.062	0.093	−0.029	−0.066	0.077	−0.036
RD	−0.361	0.047	−0.354	−0.484	0.052	−0.415	−0.183	0.047	−0.179
PC	–	–	–	–	–	–	0.368	0.040	0.421
R^2^	0.127			0.180			0.273		
Adjusted R^2^	0.121			0.174			0.266		
F	20.191			30.271			38.798		

RD, Relative deprivation; PC, Psychological capital; B, unstandardized coefficient; SE, standard error; β, standardized coefficient.

Bootstrap analysis was further conducted to test the mediating effect after controlling for gender and age. Relative deprivation was negatively associated with psychological capital (estimate = −0.484, *p* < 0.001), whereas psychological capital was positively associated with physical activity participation (estimate = 0.368, *p* < 0.001). The direct effect of relative deprivation on physical activity participation remained significant (estimate = −0.183, *p* < 0.001), and the indirect effect also remained significant (estimate = −0.178, 95% CI [−0.235, −0.127]). Because the 95% confidence interval of the indirect effect did not include zero, psychological capital continued to show a significant partial mediating role after controlling for gender and age (Table 16).

**Table 16:** Bootstrap results of the mediation effect after controlling for gender and age.

Effect	Path	Effect estimate	SE	95% CI	z/t	p-value	Result
Indirect effect	RD → PC → PAP	−0.178	0.028	[−0.235, −0.127]	−6.453	<0.001	Partial mediation
Path a	RD → PC	−0.484	0.052	[−0.586, −0.381]	−9.311	<0.001	Significant
Path b	PC → PAP	0.368	0.040	[0.289, 0.448]	9.094	<0.001	Significant
Direct effect	RD → PAP	−0.183	0.047	[−0.275, −0.090]	−3.882	<0.001	Significant
Total effect	RD → PAP	−0.361	0.047	[−0.453, −0.269]	−7.701	<0.001	Significant

RD, Relative deprivation; PC, Psychological capital; PAP, Physical activity participation. Gender and age were included as control variables. SE, standard error; CI, confidence interval. The mediation effect was tested using Bootstrap resampling.

## 5 Discussion

This study examined the relationships among relative deprivation, psychological capital, and physical activity participation among university students and tested the mediating role of psychological capital. Drawing on social comparison theory and conservation of resources theory, the study explored whether relative deprivation, as a negative social comparison experience, was associated with lower positive psychological resources and lower physical activity participation.

The findings showed that relative deprivation was negatively associated with both physical activity participation and psychological capital, whereas psychological capital was positively associated with physical activity participation. Mediation analysis further indicated that psychological capital partially mediated the association between relative deprivation and physical activity participation. The core paths and indirect association remained significant after controlling for gender and age, providing additional support for the consistency of the observed associations.

### 5.1 Relative deprivation as a negative factor associated with physical activity participation

The results showed a significant negative association between relative deprivation and physical activity participation among university students. This finding suggests that students who perceive themselves as disadvantaged through social comparison may be less likely to engage in physical activity. This result is consistent with previous studies linking socioeconomic inequality and perceived disadvantage to disparities in sports and physical activity participation.

Physical activity participation is not determined solely by personal interest; rather, it is influenced by multiple factors, including economic conditions, access to exercise resources, transportation convenience, and peer environments. Evidence from adolescent populations has shown that young people from low-income families participate in physical activity on fewer days per week and have access to a more limited range of sports activities, suggesting that disparities in resources and opportunities may directly constrain individuals’ possibilities for sports participation (Schelleman-Offermans and Massar, 2020).

Tandon et al. (2021) further reported that financial costs, sports equipment, transportation conditions, and insufficient community sports facilities were important barriers to physical activity participation among low-income adolescents. For university students, similar disparities in resources may also be subjectively amplified through social comparison. When individuals perceive themselves as inferior to their peers in terms of exercise conditions, physical ability, equipment, or social resources, physical activity may no longer be viewed merely as a health-related behavior but may instead become a comparative context in which perceived disadvantages are easily exposed. In this way, relative deprivation may be associated with lower physical activity participation, as perceived disadvantage may reduce individuals’ willingness or opportunities to engage in physical activity (Tandon et al., 2021).

This association may reflect not only external resource constraints but also individuals’ psychological evaluations of their own circumstances. Individuals from socioeconomically disadvantaged groups may recognize the health, social, and enjoyment-related value of physical activity, yet their participation may still be constrained by factors such as financial limitations, fatigue, health conditions, and other barriers (Richard et al., 2022). Instead, it may reflect the combined influence of real-life barriers and a subjective sense of disadvantage. The observed association between relative deprivation and physical activity participation provides a psychological perspective that may complement this view.

From a psychological perspective, previous theoretical perspectives suggest that relative deprivation may be associated with experiences of frustration, perceived unfairness, and helplessness. These negative experiences may be associated with lower levels of self-efficacy, sense of purpose, and behavioral control. Guo et al. (2024) found in a university student sample that relative deprivation was significantly negatively associated with psychological well-being, and that self-efficacy and self-control played important roles in this relationship (Guo et al., 2024). For physical activity participation, self-efficacy and self-control are also important psychological foundations for maintaining exercise behavior. When university students experience a long-term sense of relative disadvantage, their ability to actively participate in physical activity, adhere to exercise plans, and cope with exercise-related setbacks may be weakened.

Related research has also suggested close associations among physical activity, positive psychological resources, and relative deprivation. Higher levels of physical activity may reduce mental health risks among adolescents by enhancing psychological capital and decreasing relative deprivation (Gao et al., 2024). Although this study mainly focused on the protective role of physical activity in mental health, its findings also indirectly suggest that relative deprivation is not an isolated negative emotion but is closely related to individuals’ positive psychological resources and health-related behaviors. The present study further extends this perspective by showing from the reverse pathway that relative deprivation itself may also serve as an important psychological barrier to physical activity participation.

In sports contexts, comparison is particularly common. Physical performance, sports skills, fitness levels, and peer evaluation may all serve as important reference points through which university students assess themselves. If individuals continually perceive themselves as lagging or inadequate in such comparisons, physical activity may be experienced as a source of pressure rather than as an opportunity for relaxation, social interaction, or self-improvement. Research on competitive orientation and relative deprivation in sport-related contexts also suggests that individuals’ interpretations of competition and comparison situations may influence their exercise experiences and behavioral persistence (Zhang and Lei, 2026).

### 5.2 Relative deprivation as a negative factor associated with psychological capital

This study found that relative deprivation was significantly and negatively associated with psychological capital, indicating that perceived disadvantage, unfairness, and a sense of falling behind experienced by university students in social comparison were linked to lower levels of positive psychological resources. This finding is consistent with conservation of resources theory, which suggests that when individuals continuously perceive themselves as being in a disadvantaged situation, their psychological energy may be depleted, which may be associated with lower levels of positive psychological qualities such as self-efficacy, hope, resilience, and optimism (Lee et al., 2022).

Previous studies have shown that relative deprivation may further affect health outcomes through the depletion of psychological capital. For example, in research on health inequality among rural residents, relative deprivation was found to significantly reduce psychological capital, and psychological capital mediated the relationship between relative deprivation and health inequality. This indirect association suggests that psychological capital may represent a potential psychological pathway linking relative deprivation and physical activity participation (Tao et al., 2022).

Li C. et al. (2026) and Li F. et al. (2026) further explained this process from the perspective of ego depletion. Relative deprivation may consume individuals’ psychological energy, making it more difficult for them to maintain positive cognitive evaluations and behavioral regulation. When university students repeatedly feel that they are “inferior to others” or “unable to change their current situation,” their sense of hope and perceived control may decline, and their capacity to cope with difficulties may also be weakened (Li F. et al., 2026). Therefore, continuous psychological strain may represent one possible pathway associated with the relationship between relative deprivation and lower levels of psychological capital.

The relationship between relative deprivation and psychological capital can also be understood as a contrast between “negative comparison” and “positive resources” (Dóci et al., 2023). Higher levels of psychological capital usually indicate stronger self-efficacy, optimistic expectations, a clearer sense of goal pathways, and greater psychological resilience. In contrast, relative deprivation tends to place individuals in a psychological state characterized by perceived unfairness, helplessness, and a reduced sense of self-worth (Peng et al., 2019). Physical activity, psychological capital, and relative deprivation are closely connected. Higher psychological capital is often accompanied by lower relative deprivation, and these factors may jointly contribute to the formation of health-related behaviors and mental health outcomes (Gao et al., 2024).

Hope, as an important dimension of psychological capital, may represent a psychological resource associated with the relationship between relative deprivation and physical activity participation. Individuals with higher levels of hope may be more likely to maintain adaptive behavioral patterns when experiencing feelings of relative deprivation (Keshavarz et al., 2021). This finding suggests that psychological capital may represent an important psychological resource associated with the relationship between relative deprivation and physical activity participation. However, the cross-sectional design does not allow conclusions regarding whether psychological capital changes in response to relative deprivation or whether it protects individuals from its potential consequences.

Similarly, other positive psychological resources, such as growth mindset, may also be associated with individuals’ responses to relative deprivation and their physical activity participation. Relative deprivation may lead individuals to believe that their current circumstances are difficult to improve, thereby reducing their motivation for self-improvement. However, when individuals believe that their abilities and circumstances can be changed through effort, their willingness to act and motivation for development may be preserved (Liu et al., 2025).

### 5.3 Psychological capital as a positive factor associated with physical activity participation

This study found that psychological capital was significantly and positively associated with physical activity participation among university students, indicating that individuals with more abundant positive psychological resources are more likely to actively participate in and persist with physical activity. Psychological capital comprises self-efficacy, hope, resilience, and optimism, and these resources may help university students enhance exercise confidence, develop health-related goals, and maintain behavioral persistence when facing fatigue, time pressure, or short-term setbacks (Newman et al., 2014).

This finding is generally consistent with previous research. Psychological capital may not only be directly associated with physical activity participation among university students but may also be related to the formation of health-related behaviors by enhancing health awareness and health motivation (Huang et al., 2025). From the perspectives of health beliefs and self-determined motivation, students with higher levels of psychological capital may be more likely to recognize the health value of physical activity and to internalize external health requirements as intrinsic motivation (Luthans et al., 2006). Therefore, for these students, physical activity is not merely a task to be completed passively, but an active choice related to self-development, health management, and a positive lifestyle.

Xiong et al. (2025) also indicated that psychological capital mediated the relationship between social support and physical exercise behavior among university students, with self-efficacy playing a particularly important role in the initial stage of exercise participation. Students with higher self-efficacy are more likely to believe that they can complete exercise tasks and are better able to overcome barriers such as academic pressure, lack of time, and exercise-related difficulties (Xiong et al., 2025).

Previous research has suggested that psychological capital and physical activity participation may be mutually reinforcing over time. Positive psychological resources can promote individuals’ participation in physical activity, while physical activity itself may further enhance psychological capital, thereby forming a positive cycle (Xue et al., 2025). Previous studies have also shown that psychological capital may play an important role in the process through which physical activity influences mental health by helping individuals buffer negative cognitions, strengthen emotional regulation, and improve social adaptation (Deng and Wang, 2024). Therefore, psychological capital may represent an important psychological resource associated with physical activity participation and the psychological benefits derived from engaging in physical activity.

However, the positive association between psychological capital and physical activity participation may depend on contextual conditions. Environmental barriers, including limited exercise facilities, economic constraints, or restricted opportunities for physical activity, may influence how psychological resources are associated with individuals’ participation behaviors (Sheng et al., 2025). This suggests that although psychological capital is positively associated with physical activity participation among university students, the relationship between psychological resources and physical activity participation may be influenced by external contextual conditions.

### 5.4 Psychological capital as a mediating mechanism in the association between relative deprivation and physical activity participation

This study further showed that psychological capital partially mediated the association between relative deprivation and physical activity participation. This finding suggests that relative deprivation may be linked to lower physical activity participation not only directly but also indirectly through lower psychological capital.

This finding is generally consistent with conservation of resources theory. Relative deprivation, as a persistent negative social comparison experience, may be associated with increased psychological strain when individuals continuously focus on the gap between themselves and others, thereby increasing psychological consumption. Previous research has shown that relative deprivation can further influence health inequality by reducing psychological capital. In particular, the mediating role of psychological capital appears to be more evident in pathways related to mental health (Tao et al., 2022). This suggests that psychological capital may represent a potential explanatory pathway in the association between relative deprivation and health-related behavioral outcomes.

The ability of psychological capital to play this mediating role is closely related to its internal structure. Self-efficacy influences whether individuals believe they can complete exercise tasks; hope helps individuals set goals and identify pathways to achieve them; resilience enables individuals to recover and persist when facing fatigue, setbacks, and time pressure; and optimism helps individuals expect positive outcomes from physical activity (Nolzen, 2018). Psychological capital has been found to mediate the relationship between social support and physical exercise behavior among university students, suggesting that external support often needs to be transformed into actual exercise behavior through positive psychological resources (Xiong et al., 2025). Similarly, psychological resilience may promote adolescents’ sports participation by enhancing exercise motivation, further indicating that positive psychological resources are an important foundation for maintaining physical activity behavior (Hu et al., 2025).

From this perspective, the negative association between relative deprivation and physical activity participation may not simply reflect a “lack of interest,” but may instead be related to behavioral withdrawal when psychological resources are perceived as insufficient. When individuals perceive their circumstances as difficult to change, their motivation for self-improvement and willingness to act often decline. Relative deprivation may also weaken individuals’ positive psychological and behavioral functioning through ego depletion (Li F. et al., 2026). Therefore, a decline in psychological capital may make it more difficult for university students to view physical activity as an achievable, sustainable, and meaningful behavioral choice. Related research has also suggested close associations among physical activity, psychological capital, and relative deprivation, indicating that these factors may jointly contribute to the formation of health-related behaviors and mental health outcomes (Gao et al., 2024).

### 5.5 Limitations and future directions

Although this study provides evidence for the mediating role of psychological capital in the association between relative deprivation and physical activity participation, several limitations should be acknowledged.

First, this study used a cross-sectional design; therefore, causal inferences about the relationships among relative deprivation, psychological capital, and physical activity participation cannot be established. Although the structural equation model and bootstrap mediation analysis supported the hypothesized associations, alternative explanations cannot be completely ruled out. For example, lower physical activity participation may also be associated with reduced psychological capital, or individuals with lower psychological capital may perceive social comparisons more negatively and report higher levels of relative deprivation. Future longitudinal or experimental studies are needed to further examine the temporal order and potential causal relationships among these variables.

Second, the study relied on self-reported questionnaire data, which may be affected by recall bias and social desirability bias. In particular, physical activity participation was measured using subjective reports rather than objective behavioral indicators. Future research could combine questionnaire measures with wearable devices, mobile activity records, or third-party assessments to obtain more accurate estimates of physical activity participation.

Third, the sample was drawn from a single university in Jiangsu Province, which may limit the generalizability of the findings to students from other regions or types of institutions. University students may differ in relative deprivation, psychological capital, and physical activity participation according to socioeconomic background, grade level, institutional context, and cultural environment. Future studies should include multi-site samples from different regions and institution types to improve external validity.

Fourth, although gender and age were controlled in the model and no significant gender differences were found in the main variables, other important background factors were not included, such as grade level, academic major, family socioeconomic status, body mass index, physical education requirements, and previous exercise experience. These factors may influence both psychological resources and physical activity participation. Future research should include these potential confounders at the design stage and control for them in model testing.

Finally, qualitative methods such as interviews or focus groups could be used to explore how university students experience relative deprivation in campus life and how these experiences shape their motivation for physical activity participation. Such evidence would help clarify the psychological and contextual pathways underlying the observed associations and inform more targeted intervention strategies.

### 5.6 Theoretical and practical implications

This study contributes to the literature by shifting attention from positive predictors of physical activity participation to the potential inhibitory role of negative social comparison experiences. Previous studies on university students’ physical activity have often emphasized social support, exercise motivation, self-efficacy, and health awareness. By incorporating relative deprivation into the explanatory framework, this study suggests that insufficient physical activity participation may also be associated with perceived disadvantage, unfairness, and psychological resource depletion generated through social comparison.

The study also provides empirical support for the mediating role of psychological capital and for the application of conservation of resources theory to university students’ health-related behavior. Theoretical perspectives suggest that relative deprivation may be linked to reduced psychological resources, whereas self-efficacy, hope, resilience, and optimism may serve as psychological factors associated with exercise confidence, health-related goal setting, and physical activity participation. Thus, the pathway of relative deprivation, psychological capital, and physical activity participation offers a useful explanation for how negative social comparison experiences may be linked to health-related behavior.

The findings also have practical implications. When promoting physical activity participation among university students, universities should not rely solely on sports facilities, curriculum arrangements, or activity promotion. Attention should also be paid to students who may experience relative deprivation in campus life. For students who perceive disadvantages in economic conditions, physical ability, sports skills, or social resources, physical activity may become a context of comparison and pressure rather than a health-promoting behavior. Therefore, university physical activity programs should minimize excessive competition and overt comparison and create inclusive, supportive, and low-pressure exercise environments.

Enhancing psychological capital may also provide an important pathway for promoting physical activity participation. Universities can strengthen students’ confidence and persistence through exercise self-efficacy training, goal-setting guidance, peer support, positive feedback, and resilience-building programs. For students with higher levels of relative deprivation, strengthening hope, optimism, and coping ability may help reduce the negative influence of social comparison on physical activity participation. Therefore, physical activity promotion should address both external exercise conditions and students’ internal psychological resources.

## 6 Conclusion

This study examined the association between relative deprivation and physical activity participation among university students, with a particular focus on the mediating role of psychological capital. Relative deprivation was negatively associated with both physical activity participation and psychological capital, whereas psychological capital was positively associated with physical activity participation. Mediation analysis showed that psychological capital partially mediated the association between relative deprivation and physical activity participation. These findings suggest that relative deprivation may be linked to lower physical activity participation partly through lower positive psychological resources, including self-efficacy, hope, resilience, and optimism.

The findings suggest that promoting physical activity participation among university students should not depend only on improving sports facilities or optimizing curriculum arrangements. Universities should also consider students’ experiences of relative deprivation arising from social comparison and should strengthen positive psychological resources. Specifically, efforts to enhance exercise self-efficacy, hope, resilience, and optimistic health beliefs may help reduce the adverse association between relative deprivation and physical activity participation. Overall, this study highlights psychological capital as a potential statistical mediator in the association between relative deprivation and physical activity participation and provides implications for building a more positive, equitable, and supportive campus exercise environment.

## Data Availability

The raw data supporting the conclusions of this article will be made available by the authors, without undue reservation.
